# The interplay of glucose-dependent insulinotropic polypeptide in adipose tissue

**DOI:** 10.1530/JOE-23-0361

**Published:** 2024-04-27

**Authors:** Samrin Kagdi, Sulayman A Lyons, Jacqueline L Beaudry

**Affiliations:** 1 Department of Nutritional Sciences, Temerty Faculty of Medicine, University of Toronto, Toronto, Ontario, Canada

**Keywords:** brown adipose tissue, energy expenditure, energy metabolism, glucagon-like peptide 1, glucose control, glucose-dependent insulinotropic polypeptide, insulin sensitivity, lipid metabolism obesity, resting energy expenditure, type 2 diabetes mellitus, white adipose tissue

## Abstract

Adipose tissue was once known as a reservoir for energy storage but is now considered a crucial organ for hormone and energy flux with important effects on health and disease. Glucose-dependent insulinotropic polypeptide (GIP) is an incretin hormone secreted from the small intestinal K cells, responsible for augmenting insulin release, and has gained attention for its independent and amicable effects with glucagon-like peptide 1 (GLP-1), another incretin hormone secreted from the small intestinal L cells. The GIP receptor (GIPR) is found in whole adipose tissue, whereas the GLP-1 receptor (GLP-1R) is not, and some studies suggest that GIPR action lowers body weight and plays a role in lipolysis, glucose/lipid uptake/disposal, adipose tissue blood flow, lipid oxidation, and free-fatty acid (FFA) re-esterification, which may or may not be influenced by other hormones such as insulin. This review summarizes the research on the effects of GIP in adipose tissue (distinct depots of white and brown) using cellular, rodent, and human models. In doing so, we explore the mechanisms of GIPR-based medications for treating metabolic disorders, such as type 2 diabetes and obesity, and how GIPR agonism and antagonism contribute to improvements in metabolic health outcomes, potentially through actions in adipose tissues.

## Introduction

Glucose-dependent insulinotropic polypeptide (GIP) is secreted as a 42-amino acid peptide from the K cells of the upper small intestine in response to meal ingestion (Ugleholdt *et al.* 2006). Initially identified as gastric inhibitory polypeptide because high dose administration reduced gastric motility (Brown & Pederson 1970), GIP was renamed because its leading mode of action after a glucose bolus is to potentiate pancreatic β-cell insulin secretion through downstream signaling of the GIP receptor (GIPR) (Dupre *et al.* 1973) (reviewed in Finan *et al.* (2016)). Transcripts and translated proteins of GIP have also been detected in the brain and pancreatic α cells in mouse and human islets. Dipeptidyl peptidase-4 (DPP-4) degrades GIP in rodents (Kieffer *et al.* 1995) and humans (Mentlein *et al.* 1993), yielding a truncated and inactive version of the peptide.

The function of GIP is now known to extend beyond its role in the pancreas. The GIPR is found in the pancreas, bone, cardiomyocytes, brain, gastrointestinal (GI) tract, endothelial and neuronal cells, and adipose tissue (AT) (Seino *et al.* 2010) but not in the whole skeletal muscle or liver (Beaudry *et al.* 2019) (reviewed in Hammoud & Drucker (2022)). Recently, techniques using *in situ* hybridization detect mRNA transcripts in isolated fibro-adipogenic progenitors in mouse tibialis anterior muscle (Takahashi *et al.* 2023), but the GIPR protein remains to be confirmed in this tissue with the lack of a validated antibody. GIP increases whole-body lipid oxidation, increases AT blood flow and lipid uptake, reduces food consumption via signaling actions in the brain, and reduces body weight. Altogether, GIPR agonists have become an attractive pharmacotherapy agent for treating type 2 diabetes mellitus (T2DM) and potentially inducing weight loss in obese patients, like glucagon-like peptide-1 receptor (GLP-1R) agonists (Finan *et al.* 2016, Samms *et al.* 2020). In addition, GIPR agonists may reduce gastrointestinal issues often observed with the intake of medicines containing GLP-1R (Borner *et al.* 2021). As GLP-1R is expressed in the same tissues listed for GIPR, except for bone and AT (McLean *et al.* 2021), the link between improvements in metabolic outcomes and GIP may be driven through AT.

Humans have different types of AT responsible for regulating whole-body glucose and fatty acid (FA) metabolism, including white adipose tissues and brown adipose tissues (WAT and BAT, respectively) (Choe *et al.* 2016). WAT is responsible for storing energy in the form of intracellular triglycerides (TGs) and releasing energy in the form of FAs to other tissues in the body, while BAT is responsible for oxidizing metabolic substrates to generate heat (Cannon & Nedergaard 2004). Increased BAT recruitment and activity protect rodents from body weight gain, and when BAT is present in adult humans, it has been linked with a lower risk of visceral WAT accumulation, T2DM, cardiovascular disease, and dyslipidemia (Becher *et al.* 2021, Wibmer *et al.* 2021). However, we still do not know whether BAT is a protective organ in humans against chronic metabolic disease. Dysfunctional AT may cause metabolic health impairments and lipid spillover in the body, which may lead to lipid accumulation in non-AT, leading to obesity-related insulin resistance (Goossens & Blaak 2015) and low-grade chronic inflammation (Hu *et al.* 2018). The role of AT in regulating whole-body energy homeostasis makes understanding how therapeutic treatments impact AT function imperative for drug design.

Revisiting the physiological role of GIP in energy metabolism exposes unresolved questions regarding GIP and its self-governing physiological function apart from GLP-1. There are now several studies investigating the independent effects of GIP on obesity models in rodents and humans and how it appears to modulate glucose and lipid metabolism, as well as insulin signaling at the level of the AT. Future research may also allow the possibility of determining any sex-specific differences. This review presents current knowledge on the actions of GIP in both WAT and BAT, in cellular, rodent, and human studies. As it remains unclear whether GIPR agonism or antagonism promotes healthy AT function, this review also discusses what is known about activation and inhibition of GIPR at the AT level and its potential benefits for whole-body energy metabolism, affecting both health and disease.

## White adipose tissue

### *In vitro* effect of GIP

**Figure 1:**
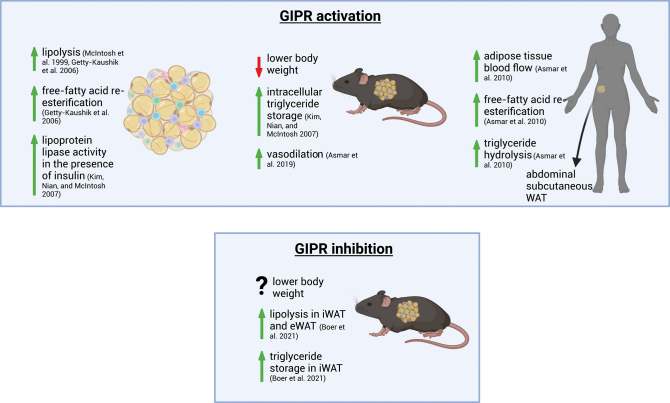
Effects of GIPR activation and inhibition in cells, and WAT from rodents and humans. GIP receptor (GIPR) is expressed in pericytes, endothelial cells, macrophages, and mesothelial cells in white adipose tissue (WAT) (Hammoud & Drucker 2022). GIPR agonism has shown an increase in lipolysis, free-fatty acid re-esterification, and lipoprotein activity *in vitro* (Kim *et al.* 2007, McIntosh *et al.* 1999, Getty-Kaushik *et al.* 2006). Adipose tissue blood flow, triglyceride lipolysis, and free-fatty acid re-esterification have increased in humans with GIP treatment (Asmar *et al.* 2010). Both GIPR activation and inhibition show a reduction in body weight *in vivo*, but reducing GIPR signaling in the whole animal increases lipolysis in iWAT and eWAT and TG storage in iWAT (Boer *et al.* 2021). Created in BioRender.

Previous studies have demonstrated that GIP administration contributes to changes in WAT lipid metabolism (Fig. 1), although more information is needed on GIPR expression in non-adipocyte fractions of WAT (Campbell *et al.* 2022). Previous work highlights GIP’s role in lipid uptake and storage in differentiated white adipocytes. For example, GIP treatment (100 nM) in the presence of insulin (1 nM) increases lipoprotein lipase (LPL) activity in both differentiated murine-derived WAT (3T3-L1) cells and subcutaneous human adipocytes (Kim *et al.* 2007), leading to increases in TG stores. The increase in LPL-mediated TG uptake was attributed to the upregulation of the protein kinase B activity, which mediates downstream proteins such as liver kinase B1 and AMP-activated protein kinase. These findings indicate that, in the presence of insulin, GIP increases LPL activity, likely aiding with exogenous TG uptake and storage in WAT. However, it has also been shown that GIP dose dependently increases LPL activity in differentiated 3T3-L1 adipocytes in the absence of insulin (Miyawaki *et al.* 2002). Therefore, there are opposing findings on whether GIP, in the presence or absence of insulin, improves LPL activity.

**Figure 2:**
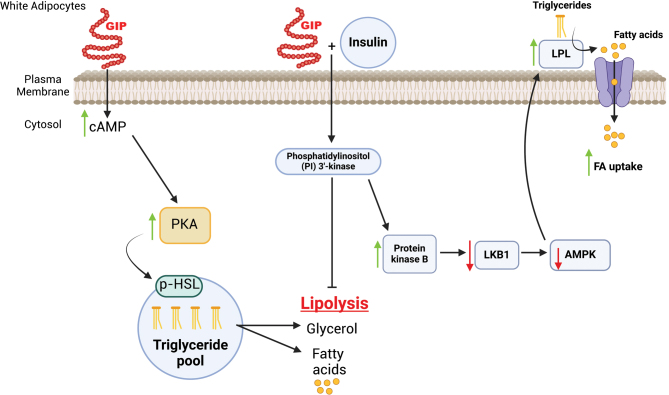
GIP-induced lipolysis and insulin signaling in white adipocytes. Exogenous administration of GIP to 3T3-L1 cells increases lipolysis through the cAMP pathway (Getty-Kaushik *et al.* 2006, McIntosh *et al.* 1999). Pre-incubation with insulin in differentiated 3T3-L1 cells followed by GIP exposure inhibits the lipolytic effect of GIP. Evidence suggests that GIP treatment in the presence of insulin increases the phosphorylation of protein kinase B (PKB) and decreases the phosphorylation of LKB1 and AMP-activated protein kinase (AMPK), causing LPL activation and TG accumulation in differentiated 3T3-L1 cells (Kim *et al.* 2007). The primary role of AMPK is to stimulate the pathway which increases lipid oxidation and suppresses lipogenesis and lipolysis, while LKB1 is responsible for phosphorylating AMPK (Daval *et al.* 2006). Created in BioRender.

GIP also plays a role in lipid breakdown in WAT. Exposure to GIP concentrations over the range of 0.1 and 1000 nM, for 4 h in 3T3-L1 adipocytes (McIntosh *et al.* 1999), and 1, 10, and 100 nM of GIP for 1 h in isolated primary rat adipocytes (Getty-Kaushik *et al.* 2006), produced higher lipolytic rates compared to basal levels, leading to increased free-fatty acid (FFA) re-esterification (Getty-Kaushik *et al.* 2006). In addition, 3T3-L1 cells treated with GIP concentrations of 1, 10, and 100 nM increased cyclic AMP (cAMP) levels, indicating that GIP-stimulated lipolysis is due to changes in cAMP activity (McIntosh *et al.* 1999), which may be contributing to the stimulation of lipolysis. The cAMP pathway plays a crucial role in GIP-induced lipolysis, as demonstrated by the use of an adenylyl cyclase inhibitor MDL 12330A (10^−4^ M), which reduced cAMP and glycerol levels in the presence of GIP (0−100 nM) (McIntosh *et al.* 1999). When 3T3-L1 cells are pre-incubated with insulin, followed by GIP exposure, the lipolytic effect of GIP is completely inhibited. The pre-incubation addition of wortmannin (a potent inhibitor of phosphatidylinositol 3-kinase, PI3K, 10^−7^ M) to insulin restores GIP’s lipolytic effects. These findings suggest that insulin is dependent on the PI3K mechanism to inhibit GIP-induced lipolysis (Fig. 2). On the contrary, while GIP (10 nM) or insulin alone (10 µU/mL) increased lipolysis and FFA re-esterification compared to basal levels (Getty-Kaushik *et al.* 2006), concurrent administration of GIP (10 nM) and insulin (10 µU/mL) to 3T3-L1 cells (i.e. no pre-incubation) did not change rates of glycerol and FFA release. The regulatory role of GIP for white adipocyte lipolysis and FFA re-esterification appears to be dictated by the presence of insulin, requiring further investigation into how GIP and insulin impact signaling mechanisms in WAT.

Research also shows that GIP has anti-lipolytic actions in adipocytes. Isoproterenol (β_1,2_-adrenergic receptor agonist; 1 μM) increased lipolysis in isolated rat adipocytes (Getty-Kaushik *et al.* 2006), but the addition of GIP to isoproterenol significantly decreased lipolysis. Moreover, the addition of a GIPR antagonist (ANTGIP) to GIP and isoproterenol resulted in a restoration of the lipolytic response, suggesting that GIPR agonism may play an inhibitory role in stimulated lipolysis in white adipocytes.

Dose and media conditions play important roles in understanding the effects of GIP on lipid metabolism in AT, where GIP at concentrations of 1–10 nM has a strong lipolytic effect on adipocytes under low insulin conditions in the context of *in vitro* murine-derived cell culture models. Moreover, dosing typically conducted in cell culture models that report changes in lipid flux within the adipocytes would be defined in the pharmacological range, as post prandial GIP levels are found to be ~400–600 pg/mL in rodents (Campbell *et al.* 2016) and ~1000–1500 pg/mL in humans (Nauck *et al.* 1986).

In addition to its impact on WAT and lipid uptake, GIP has also been shown to promote an increase in the translation of glucose transporter type 4 (GLUT4: a protein that aids in glucose uptake in AT in response to insulin) in 3T3-L1 adipocytes wild-type (WT) GIPR, or expressing E354Q GIPR, a mutation in the GIPR gene, a variant associated with insulin resistance, T2DM, and cardiovascular risk in humans. This effect suggests that the downstream signaling pathways activated by GIP are not altered in the presence of the E354Q variant (Mohammad *et al.* 2014), although the variant has been shown to result in the downregulation of GIPR from the plasma membrane after GIP stimulation.

### *In vivo* effect of GIP in rodents

Although the presence of the GIPR within the whole AT implies that GIP may be exerting specific effects on WAT function, the exact mechanisms remain unknown. Gain-of-function studies with GIP treatment demonstrate increased WAT vasodilation and lipid clearance (Asmar *et al.* 2019), while reducing body weight, food intake, and fat mass, but not lean mass (Zhang *et al.* 2021, Liskiewicz *et al.* 2023). Furthermore, elevated LPL activity and intracellular TG storage in epididymal WAT (eWAT) were observed in obese Vancouver diabetic fatty (VDF) rats compared to controls after 2 weeks of treatment with GIP (10 pmol/kg/min) (Kim *et al.* 2007). These findings suggest that fat storage in rodents is affected by LPL activity, but it is unclear if insulin or other hormones are necessary facilitators.

Whole-body overexpression of GIP levels (using GIP-transgenic, heterozygous mice) has been used to evaluate fat development (Kim *et al.* 2012). GIP-transgenic mice gained less body weight than WT littermate control mice fed a high-fat diet (HFD) and were more insulin-sensitive compared to WT littermates, independent of adipocyte epididymal size. GIP-transgenic mice also exhibited a lower overall fat percentage and adiposity index compared to WT mice. The reduced adiposity observed in GIP-transgenic mice was associated with decreased food intake independent of lower energy expenditure. However, these mice did exhibit reduced physical activity, possibly related to the overexpression of hypothalamic GIP levels. On the contrary, GIPR-deficient mice have shown increased energy expenditure associated with increases in locomotor activity (Hansotia *et al.* 2007), trends to exhibit lower anxiety-like behavior, improved exploration, spatial learning, and memory ability (Takahashi *et al.* 2023). These findings suggest a complex interplay between GIP signaling and behavioral and cognitive functions and their effects on whole-body metabolism. GIP-transgenic mouse eWAT also exhibited reduced mRNA levels of genes related to FA synthesis, mitochondrial biogenesis and function, and gene expression levels relating to inflammation and insulin responses. Protein expression for insulin receptor 1 decreased, while that for insulin receptor 2 increased in the eWAT of GIP-transgenic mice, maintaining insulin sensitivity. Recent studies show that long-acting GIPR (LA-GIPR) agonism signaling pathways in the central nervous system regulate food intake, body weight, fat mass, and glucose homeostasis (Zhang *et al.* 2021). These results appear to be regulated by the hypothalamic expression of GIPR, which has recently been reported to be a contribution of the inhibitory GABAergic neuronal population (Liskiewicz *et al.* 2023). GIPR has also been found to be expressed in brain regions such as the olfactory bulb, cerebral cortex, and hippocampus, and is responsible for regulating appetite and satiety (Usdin *et al.* 1993). GLP-1R is co-expressed in some neuronal areas with GIPR such as the dentate gyrus of the hippocampus, nucleus tractus solitarius, and area postrema of the dorsal vagal complex (reviewed in Hammoud & Drucker (2022)). Therefore, the overexpression of GIP may be driven by the central effects on WAT and is less likely because of WAT’s regulation of GIP in the brain. However, it is important to consider that since WAT *Gipr* mRNA expression levels are relatively higher than those detected in the hypothalamic regions of the brain (Zhang *et al.* 2021), there may be potential cross talk between the central nervous system and WAT.

Moreover, advancements in the development of GIP agonists have enhanced our understanding of GIP’s effect on body weight and appetite regulation. GIPFA-085, GIP-D-Ala_2_, and LA-GIPR agonists have a modified amino acid sequence or N-terminus compared to the native GIP, which protects against DPP-4 cleavage (Irwin & Flatt 2009), extending the circulating half-life of GIP from 4 to 7 min (Vilsboll *et al.* 2006) to several hours in mice (i.e. 06:29 h (Samms *et al.* 2021)). The increased half-life allows more time for administered GIP to interact and exert its influence on peripheral tissues. One study demonstrated that GIPFA-085 (300 nmol/kg; half-life 6.55 h) administered to diet-induced obese (DIO) mice, decreased daily food intake on days 1–3 and significantly reduced body weight gain on days 3–12 (Han *et al.* 2023). Another study found that LA-GIPR treatment showed increased insulin sensitivity in the AT, and increased glucose uptake into WAT, but no changes in gene expression regulatory pathways were observed (Samms *et al.* 2021). On the contrary, HFD-fed mice given daily intraperitoneal injections of GIP-D-Ala_2_ (0.12 µg/g) in the last 8 weeks of a 14-week HFD showed no differences in body weight, food intake, or visceral fat weight compared to vehicle controls (Varol *et al.* 2014). However, GIP-D-Ala_2_-treated DIO mice more than doubled epididymal adipocyte size (Varol *et al.* 2014), indicating greater lipid storage capacity. Increased gene expression in *plin1*, *cidea*, *cidec*, and *pparγ* in lipid droplets in male mice epididymal fat was also observed, allowing for greater lipid deposition in AT (Varol *et al.* 2014). Overall, these findings suggest that exogenous GIP administration improves whole-body substrate deposition in WAT; however, differences in agonist structure, dose, timing of administration, and agonist half-life may all impact food intake and body weight loss. Future work should carefully consider dosage conditions when assessing the effects of GIP on AT tissue function *in vivo*.

### *In vivo* effect of GIP in humans

Studies have investigated the effects of GIP on WAT lipid metabolism in rodents but have not revealed the role of GIP action on human WAT. Researchers employing intravenous clamps to control circulating glucose and insulin levels in human subjects (Asmar *et al.* 2010, Asmar *et al.* 2014, Asmar *et al.* 2016*b*) have found that, in lean, healthy human subjects under hyperinsulinemic–hyperglycemic conditions, GIP infusion increased abdominal subcutaneous white adipose tissue blood flow (ATBF), FFA re-esterification, glucose uptake, LPL activity, and TG hydrolysis, leading to increased TG storage in the anterior, abdominal subcutaneous WAT compared to vehicle controls (Asmar *et al.* 2010). Another study found that in lean humans under hyperinsulinemic–euglycemic conditions, lipid uptake increased, and lipid breakdown decreased in the presence of GIP (Asmar *et al.* 2016*b*). These findings demonstrate that insulin facilitates GIP-mediated TG uptake into WAT and inhibits lipid breakdown through decreases in WAT lipolysis (Asmar *et al.* 2016*b*).

In contrast, under high glucose and insulin conditions, there were no differences in subcutaneous AT lipid storage among obese individuals with higher glucose excursions compared to subjects who were classified as responsive to a glucose bolus (Asmar *et al.* 2014). Additionally, FFA/glycerol ratios were higher in the impaired versus normal glucose-tolerant obese group (Asmar *et al.* 2014), indicating reduced FFA re-esterification. This likely resulted from impaired glucose uptake and inhibition of lipolysis mechanisms directly at the level of the WAT. Therefore, it may be that in the presence of insulin, GIP promotes the storage of FFAs in subcutaneous WAT instead of remaining in the circulation of healthy individuals.

Many studies suggest that GIP’s effects *in vivo* on AT glucose uptake, TG hydrolysis, and FFA re-esterification may result from GIP stimulation and elevation of circulating insulin levels (Asmar *et al.* 2017). Insulin drives nutrient storage into organs such as AT; however, GIP’s effect on energy metabolism and homeostasis is complicated and may act independently of insulin. For example, studies in healthy lean human subjects have shown a decrease in non-esterified fatty acids (NEFA) after exogenous GIP administration, with hyperinsulinemia and slight hyperglycemia (Asmar *et al.* 2017). However, when type 1 diabetes mellitus (T1DM) patients (with low insulin production) were provided a subcutaneous GIP infusion, they showed an increase in NEFA levels during the first 3 h, without changes in fasting plasma levels of C-peptide or insulin (Heimburger *et al.* 2022). GIP infusions initially lowered the respiratory exchange ratio (RER) in T1DM subjects within the first 150 min, possibly indicating changes in fuel selection, favoring lipids versus glucose (Heimburger *et al.* 2022). However, liver fat accumulation increased in GIP-treated T1DM individuals following 6 days of GIP administration. More work is needed to assess the interplay between insulin and GIP on lipid deposition. In the absence of insulin, exogenous GIP may increase circulating NEFAs (indicative of higher rates of lipolysis), but the presence of insulin with exogenous GIP promotes healthy lipid deposition into AT. Clinical trials are currently exploring LA-GIPR agonists (Muller *et al.* 2022) to determine their effects on body weight loss, T2DM, and energy metabolism, and whether GIP actions are dependent or independent of insulin levels and/or insulin sensitivity.

In the fasted state, GIP treatment plays no role in WAT lipid metabolism (Asmar *et al.* 2010), suggesting that glucose or insulin levels may be required for GIP to regulate lipid metabolism in humans. These impairments in GIP-induced WAT metabolism are more revealing in obese subjects who have reduced insulin sensitivity and reduced capacities to convert glucose into glycerol (Asmar *et al.* 2016*a*). Individuals who lose weight and reinstate insulin sensitivity may also improve GIP signaling at the level of the subcutaneous AT (Asmar *et al.* 2016*a*). However, it is unclear whether there are differences between subcutaneous and visceral AT and GIP sensitivity. These conclusions are confirmed where GIP infusions alone increased TG uptake and decreased FFA output and FFA/glycerol ratios compared to the effects of GIPR antagonist (GIP (3–30)NH_2_), which decreased TG uptake and increased white ATBF in lean subjects (Asmar *et al.* 2017). These findings indicate that GIP can contribute to lower circulating lipid levels by increasing TG offloading and reducing lipolysis in healthy human WAT. Adipogenic effects of GIP have also been assessed in T2DM obese individuals under fasting conditions, where plasma NEFA concentrations decreased with GIP infusion while increasing subcutaneous AT TGs (Thondam *et al.* 2017). This effect was linked to insulin sensitivity of the individual. GIP-induced TG uptake into AT may be dependent on the level of AT insulin resistance, which can enhance obesity and T2DM conditions.

Whether GIP sensitivity changes in WAT under hyperglycemic conditions, similar to pancreatic beta cells (Vilsboll *et al.* 2002), remains an area of investigation. However, it is important to note that disruption in GIP signaling observed in individuals with T2DM is linked with increasing measurements of body mass index (reviewed in Finan *et al.* (2016)). Thus, GIP sensitivity in WAT may diminish under conditions of insulin resistance or hyperglycemia, leading to impaired fat metabolism. However, the exact mechanisms underlying this phenomenon are not clearly understood.

Besides GIP’s effects on glucose and lipid metabolism, GIP may provide additional benefits to WAT function via ATBF. GIP administration to lean, healthy humans during hyperinsulinemia causes a four-fold increase in blood flow and TG clearance in subcutaneous abdominal AT when compared to saline controls (Asmar *et al.* 2019)_._ This suggests that GIP may enhance capillary recruitment in WAT, increasing blood flow and substrate transport into WAT (Tobin *et al.* 2010). Increased ATBF may result in increased interactions between circulating lipoproteins and AT LPL (Asmar *et al.* 2019), which likely promotes TG hydrolysis and TG uptake in AT. ATBF increased three-fold after 30 min during GIP infusion when subjects were exposed to hyperinsulinemic–euglycemic conditions (Asmar *et al.* 2016*b*). However, under fasted conditions, GIP has no effect on ATBF in subcutaneous abdominal WAT (Asmar *et al.* 2010), further suggesting the importance of the presence of insulin for increasing ATBF. Overall, these studies have helped gain a better understanding of the direct effects of GIP on AT in humans.

In addition to the effects of GIP on ATBF, GIP exerts several other effects on vascular functions, including antiatherogenic actions that help prevent the formation of plaques in the arteries and nitric oxide production (reviewed in Heimburger *et al.* (2020)), which regulates blood flow and vascular tone (Chen *et al.* 2008). GIP suppresses the inflammatory responses to monocytes, macrophages, and adipocytes; however, these effects have mainly been demonstrated in rodent models (Varol *et al.* 2014). There are multifaceted effects of GIP on ATBF and vascular function; however, further investigation on rodents with obesity and T2DM will help understand whether the effects of GIP are consistent across different pathological conditions.

### GIPR-based therapies and their effects on WAT

Gut-hormone therapies have become prevalent in the treatment of obesity and T2DM (Tschop *et al.* 2023). GLP-1R agonists induce satiety (Naslund *et al.* 1999), but they also can cause nausea and vomiting (Bettge *et al.* 2017) that appear to escalate with higher dose concentrations or during medication titrations (Aroda & Ratner 2011). Co-agonists combine GLP-1R agonists to lower food intake and increase satiety, and GIPR agonists to reduce incidences of adverse gastrointestinal effects, improve insulin sensitivity, and increase energy metabolism in AT.

Histological analysis of eWAT in DIO mice chronically treated with a GIPR–GLP-1R agonist showed reduced adipocyte size when compared to the use of liraglutide (GLP-1R agonist) and vehicle control (Finan *et al.* 2013). In addition, treatment with the GIPR–GLP-1R co-agonist reduced overall fat mass and body weight in a dose-dependent manner (3–30 nmol/kg) in DIO mice compared to treatment with exendin-4 (GLP-1R agonist, dosed at 10 nmol/kg and 30 nmol/kg). Adding a polyethylene glycol tail to enhance bioavailability and half-life (PEGylated GIPR–GLP-1R co-agonist) of GIPR/GLP-1R dual agonism lowered body weight by 26.9%, whereas the acylated co-agonist lowered it by 31.4%, and liraglutide by 15.6%, with all three agonists similarly lowering food intake and improving dyslipidemia and hyperglycemia (Finan *et al.* 2013). Both the PEGylated co-agonist and acylated co-agonist similarly decreased plasma leptin and TG levels compared to liraglutide and vehicle control-treated DIO mice, but this is likely driven by greater body weight loss with treatments (Finan *et al.* 2013). Liraglutide treatment increased plasma FFA levels, whereas the GIPR–GLP-1R co-agonist did not affect circulating free lipid levels. Therefore, including GIPR agonists in medications may counteract the effects of a GLP-1R-induced rise in FFA levels, but these mechanisms of action are unclear as GLP-1R is not expressed in AT. Overall, the effectiveness of GLP-1R agonists may be higher in the presence of GIPR agonists (Samms *et al.* 2020), with GIPR–GLP-1R co-agonists inducing much greater reductions in fat mass and weight loss than GLP-1R agonists alone (Frías *et al.* 2021). It is likely that combining these two peptides allows each to provide direct signaling in the respective tissue that expresses their receptor. Given the dramatic effects these peptide therapies have on reductions in body weight and fat mass, more work assessing specific actions on AT function and whole-body energy metabolism independent of lower body weight will help guide the dramatic effects of peptide therapies on reductions in body weight loss and fat mass.

Work has demonstrated that 14 days of treatment with a GIPR–GLP-1R-co-agonist (tirzepatide, 10 nmol/kg) lowered FFA, TG levels, and hepatic lipid content in obese insulin-resistant (IR) mice compared to those treated with vehicle control (Samms *et al.* 2021). Tirzepatide improved insulin-stimulated glucose disposal in several tissues, including soleus skeletal muscle, eWAT, and iWAT, more than in pair-fed mice, indicating weight-independent improvements in whole-body insulin sensitivity in obese IR mice. In addition, WT and obese IR whole-body Glp-1r^−/−^ mice given vehicle or tirzepatide for 14 days were found to have no effect on insulin-stimulated glucose uptake in subcutaneous WAT but did show an enhancement in overall glucose infusion rates and glucose uptake in eWAT. These results indicate that tirzepatide may induce an effect of glucose uptake in WAT mediated through the GIPR. Samms *et al.* (2021) also showed that a LA-GIPR agonist (300 nmol/kg; half-life 06:29 h) administered daily for 14 days increased glucose uptake and insulin sensitivity in visceral and subcutaneous WAT in HFD-fed obese IR mice. However, these changes were insufficient to change gene expression related to glucose FA metabolism in WAT. We can conclude that GIPR agonism may enhance insulin sensitivity and act within the AT, independent of the actions of GLP-1R agonism.

Combining GIPR–GLP-1R agonists results in weight loss and lower glycemic levels in rodents and obese humans with T2DM. The use of tri-agonists, which include GIPR–GLP-1R and glucagon receptor (Gcgr) agonists, shows additional substantial effects, including the prevention of body weight gain, decreased food intake, and improvements in glucose and lipid profiles that exceed those of dual or mono-agonists (Finan & Douros 2022, Bailey *et al.* 2023) in both rodents and humans (Knerr *et al.* 2022, Jastreboff *et al.* 2023, Rosenstock *et al.* 2023). Teasing apart GIP’s independent effects in experiments using tri-agonist therapy has been challenging, but it has been found that GIPR agonism alone reduced body fat by 6.4% and reduced cumulative food intake in DIO mice compared to vehicle control (Finan *et al.* 2015). After 20 days in male DIO mice, the tri-agonist (3 nmol/kg) showed itself to be more potent in decreasing body weight by 26.6%, as opposed to the GIPR–GLP-1R co-agonist (3 nmol/kg) at 15.7%. Both WT and Gipr^−/−^ HFD-fed mice treated with the tri-agonist showed similar reductions in fat mass, food intake, and body weight (Finan *et al.* 2015). However, the exact role of GIPR agonism separated from the actions of GLP-1R and Gcgr is still uncertain. Central actions of GIP may be responsible for the reduced body weight in DIO mice treated with co- and tri-agonist therapy (Samms *et al.* 2021)_._ Moreover, the exact tissue and cell type of GIP signaling in WAT (identified as pericytes (Campbell *et al.* 2022)) responsible for changes in white fat mass or adipocyte size remains to be fully characterized, but it is likely that the contributions of GIPR may work independently of GLP-1R and Gcgr actions. Lastly, the independent effects of GIP signaling with dual and/or tri-agonism is yet to be fully determined in humans. However, clinical trials are currently being conducted to determine GIP’s effect on body weight loss and glucose control.

### *In vivo* inhibition of GIPR in WAT

There have been extensive studies on the loss of function of GIPR and the effect on WAT. As GIP was classified as an obesogenic hormone (Goralska *et al.* 2018), these ideas are complemented by demonstrating that reducing *Gipr* content in the whole animal leads to a reduction in white adipocyte size and mass and overall WAT depot. However, this is only found in male mice being fed a HFD for an extended period of time of more than 20 weeks (Miyawaki *et al.* 2002). This led to the thought that perhaps the GIPR in WAT is a key component to overall body weight gain in mice. More recent research suggests that the GIPR is not expressed in white adipocytes (Campbell *et al.* 2022), posing an interesting interpretation of the data summarized below.

AT-specific GIPR knockout mice (*Gipr*^adipo−/−^) fed a HFD have greater lean mass and lower body weight loss compared to floxed GIPR (*Gipr*^fl/fl^) mice; however, no differences in subcutaneous and visceral fat masses are observed between groups, but liver volume, weight, and fat content were lower in male* Gipr*^adipo−/−^ HFD-fed mice (Joo *et al.* 2017). Furthermore, HFD-fed *Gipr*^adipo−/−^ mice showed significantly lower C-peptide and insulin levels, and body weight, with no changes in food intake, total GIP levels, and fed blood glucose levels compared to control mice (Joo *et al.* 2017). There were lower glucose excursions during glucose, insulin, and pyruvate tolerance tests in *Gipr*^adipo−/−^ compared to WT HFD-fed mice (Joo *et al.* 2017). These findings indicate that GIPR signaling in AT is independent of food intake regulation, but insulin and tissue response to glucose appear to be lower in mice without the GIPR in WAT. However, it is still not known if this effect is driven by lower body weight or circulating insulin levels. It is also important to note an important caveat of the model used to generate *Gipr*^adipo−/^^−^ mice was the *Ap2/Fabp4*-Cre promoter, which is known to drive unspecific effects in non-adipocyte tissues (Martens *et al.* 2010). Furthermore, it has been demonstrated that the insulin secretory response to GIP does increase food intake in WT mice fed a HFD, whereas this response is absent in WT mice fed a high-carbohydrate diet (Maekawa *et al.* 2018). However, these effects are not seen in whole-body GIPR-deficient mice, which show resistance to body weight gain. It remains unclear if the mechanism behind reducing GIPR action at the pancreas results in lower insulin levels that indirectly act to regulate WAT function, or if it is GIPR inhibition at the level of the whole WAT that regulates energy metabolism and insulin signaling. Moreover, the feeding centers of the brain may play a role in these effects. It has been reported that the increase in physiological GIP secretion due to overnutrition attenuates the suppressive effect on feeding by leptin in the hypothalamus (Kaneko *et al.* 2019). Thus, this may contribute to weight gain as there will be a diminished effect by the hypothalamus in controlling for food intake. As such, there may be complex mechanisms governing food intake in the hypothalamus through the interactions between leptin and GIPR, as both seem to play a role in regulating appetite and satiety. These findings suggest that GIP sensitivity in AT and pancreas, in addition to its direct effect on fat accumulation, plays a role in regulating body weight gain and food intake by GIP action in the hypothalamus (reviewed in Seino & Yamazaki (2022)).

Impaired AT function can significantly impact AT lipid metabolism, insulin sensitivity, and, ultimately, regulation of body weight (Chouchani & Kajimura 2019). Interrogation of the specific effects of AT GIPR was further demonstrated by Boer *et al.* 2021, where no differences were detected in FA oxidation and uptake in inguinal WAT (iWAT) isolated from male Gipr^−/−^ and wild-type littermates on a 45% HFD. There was, however, an increase in iWAT and eWAT lipolysis in male Gipr^−/−^ HFD-fed mice that were more exaggerated after β-adrenergic stimulation (Boer *et al.* 2021)_._ Measurements of TG storage analyzed by^ 3^H-labeled triolein in Gipr^-/-^ mice were higher in iWAT, whereas there were no differences in TG storage in BAT and eWAT. The removal of GIPR in WAT may have depot-specific effects on the regulation of AT lipolysis and lipid storage but, again, it is difficult to determine if these are due to weight loss or insulin level differences detected between WT and Gipr^−/−^ genotypes.

Similar to removing GIPR action in AT with the use of AT GIPR knockout mice models, studies have shown that GIPR antagonism lowers body weight in the face of HFD feeding (Lu *et al.* 2021). Paradoxically, both LA-GIPR agonist and muGIPR-Ab independently reduced weight gain in male DIO mice (Killion *et al.* 2018, 2020). Blocking GIPR using muGIPR-Ab did not affect food intake or FA uptake into primary mouse white adipocytes, while the LA-GIPR agonist decreased food intake from days 1 to 3 (Killion *et al.* 2020). Treatment with either LA-GIPR or muGIPR-Ab, in combination with liraglutide, a GLP-1R agonist, was better at reducing weight gain than muGIPR-Ab treatment alone in male DIO mice. Therefore, different mechanisms of action of GIPR agonist and antagonists may work independently to reduce body weight, but paired with a GLP-1R agonist, both provide integration at the level of the GIPR. It does appear that GIPR agonism results in increased levels of insulin secretion, whereas GIPR antagonism results in reduced levels of insulin (Killion *et al.* 2020), thereby suggesting a difference in plausible mechanisms of action, at least in the pancreatic beta cells. GIPR agonists have also been found to act as GIPR antagonists in WAT (lower GIPR expression than pancreatic islets), whereby GIPR agonism results in desensitization of GIPR activity (Killion *et al.* 2020). Moreover, when GIPR β-cell knockout mice (*Gipr*^βCell−/−^) were compared to *Gipr*^fl/fl^ control mice, there were no differences in weight loss effects when treated with a HFD or when administered muGIPR-Ab alone, GLP-1R agonist, or in the combination (Killion *et al.* 2018), complementing data that GIPR in WAT may be partially responsible for lower body weight, independent of insulin signaling.

In humans, the use of the GIPR antagonist, GIP(3–30)NH_2_, alone or in combination with GIP, decreases AT TG uptake (Asmar *et al.* 2017). GIP(3–30)NH_2_ (800 pmol/kg/min) was assessed in healthy individuals and showed no changes in plasma TGs, glycerol, cholesterol, and NEFA content with GIP(3–30)NH_2_ alone or with a GIPR agonist (Gasbjerg *et al.* 2018). These data coincide with findings in lean subjects, where GIP infusion increases ATBF five-fold compared to vehicle control; however, when GIP was combined with GIP(3–30)NH_2_, the increase in ATBF was attenuated (Asmar *et al.* 2017). These studies support the hypothesis that WAT GIPR agonism is responsible for ATBF and lipid uptake. While most studies have assessed GIPR antagonism in rodent models, more work is needed to further elucidate the impact of GIPR antagonists in patients with T2D and obesity.

Altogether, these studies demonstrate the ambiguity surrounding the inhibition of GIPR and promoting GIP action to treat obesity and T2DM (these topics have been extensively reviewed by Killion *et al.* (2019), Campbell (2021), and Samms *et al.* (2020)). It remains unclear how to reduce fat mass in the most effective way while providing metabolic improvements. Recent advancements suggest that combining either GIPR agonism or antagonism with a GLP-1R agonist results in significant metabolic benefits that are superior to the mono-agonism of the incretin receptors (Tschop *et al.* 2023).

## Brown adipose tissue

### *In vitro/in vivo* effects of GIP

**Figure 3:**
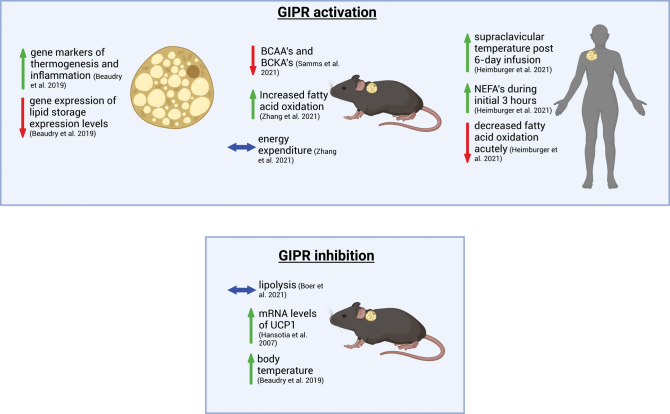
Effects of GIPR activation and inhibition in cells, and BAT from rodents and humans. GIP treatment increases gene expression in thermogenic and inflammatory pathways, whereas genes related to lipid storage are reduced in BAT cells *in vitro* (Beaudry *et al.* 2019*)*. Activation of the GIPR results in lower BCAA/BCKA ratios (Samms *et al.* 2022), decreased RER or increased FA oxidation, and no change in overall energy expenditure *in vivo* in mice (Zhang *et al.* 2021). GIP administration in humans increased supraclavicular temperature after 6 days of GIP infusion and NEFAs during the initial 3 h of treatment while acutely lowering fatty acid oxidation (Boer *et al.* 2021). GIPR BAT knock-out mice show an increase in mRNA UCP1 levels (Hansotia *et al.* 2007), decreased body weight when housed in chronic cold temperatures with no change in lipolysis. Fasted BAT GIPR knock-out mice increased body temperature in an acute cold challenge *in vivo* (Beaudry *et al.* 2019). Created in BioRender.

*GipR* mRNA levels are detected in immortalized cell lines and whole tissue explants of BAT, although they have low expression (cycle threshold levels around 29–31) (Adriaenssens *et al.* 2019, Beaudry *et al.* 2019). GIP treatment (4 h at a dose of 100 nM) in differentiated BAT cells increased gene markers of thermogenesis and inflammation, and lowered lipid storage expression levels (Beaudry *et al.* 2019). These findings indicate that GIP may regulate energy metabolism and thermogenic programming directly in BAT (Fig. 3). Acute and chronic administration of acyl-GIP (30 nmol/kg) to DIO mice showed no changes in whole-animal energy expenditure and had no effect on oxygen consumption in individual brown adipocytes (Zhang *et al.* 2021). However, acute administration of acyl-GIP (30 nmol/kg) decreased RER and increased FA oxidation, suggesting a preferential increase in whole-body lipid oxidation. Chronic acyl-GIP treatment (7 days) decreased both energy absorption and energy use, measured as ratios of food to fecal energy content (KJ/g of food), indicating reduced metabolizable energy stores in DIO mice. It remains unknown how and which tissues contribute to the GIP-induced rise in FA oxidation pathways detected in HFD obese rodents treated with exogenous GIP, but these appear to be independent of food intake and body weight lowering mechanisms of the brain (Zhang *et al.* 2021).

LA-GIPR agonist administration (300 nmol/kg; once daily for 14 days) changed regulatory genes in BAT of obese IR male mice with no changes in genes from skeletal muscle or WAT (Samms *et al.* 2021). The upregulated genes were associated with glucose oxidation, lipid, and branched-chain amino acid (BCAA) metabolism (Samms *et al.* 2021). Contributions of LA-GIPR agonists with GLP-1R may increase insulin sensitivity by improving energy metabolic pathways in BAT through upregulated gene expression. This increase in sensitivity may also be due to increased glucose uptake in visceral and subcutaneous AT independent of decreases in body weight and food intake. Moreover, treatment with tirzepatide or a LA-GIPR agonist in obese and IR mice decreased branched-chain amino acids/branched-chain keto acids (BCAAs/BCKAs) and greatly increased the expression of genes related to BCAA catabolism (Samms *et al.* 2021). Interestingly, tirzepatide appears to enhance insulin sensitivity much greater than a GLP-1R agonist alone and increases amino acid pools in BAT that phenotypically mimic thermogenically active BAT (Samms *et al.* 2022). Furthermore, obese mice given tirzepatide for 2 weeks at 10 nmol/kg stimulated the catabolism of BCAA/BCKA in BAT and increased the breakdown of BCAA by-products compared to controls and pair-feeding controls (Samms *et al.* 2022). This suggests that BAT is directly affected by tirzepatide independent of body weight loss. This work presents novel findings showing that tirzepatide improves insulin sensitivity in male DIO mice potentially through BAT-mediated mechanisms. Moreover, in recent literature, using chronic dosing of a LA-GIPR at 10 nmol/kg for 14 days showed increased glucose disposal in BAT and gene expression levels of markers in browning of WAT, lower TCA cycle flux, and mitochondrial oxidative phosphorylation pathways in BAT. This was further demonstrated by LA-GIPR treatment that provides protection against rosiglitazone-induced weight gain and systemic insulin resistance (Furber *et al.* 2023). It remains unclear if the effects of GIP *in vivo* modulate BAT energy substrate usage in humans, as no studies, at least to our knowledge, have examined GIPR expression or performed direct experiments *in vitro*/*ex vivo* experiments with GIP treatment in human BAT cells.

There is a study that has focused on the GIPR and BAT activity in men with T1DM. They were administered a 6-day subcutaneous GIP infusion (6 pmol/kg/min) and found no increase in BAT activity (supraclavicular skin temperature) after acute (120 min) treatment compared to the placebo (Heimburger *et al.* 2022). However, there was an increase in supraclavicular skin temperature post-6-day infusion, suggesting that GIP may be playing a role in regulating BAT thermogenesis in humans (Heimburger *et al.* 2022). There was a decrease in RER at 150 min, suggesting that GIP infusion increases FA oxidation, at least for a short period after administration as this effect was not sustained (Heimburger *et al.* 2022). Moreover, no changes were observed in TG and glycerol concentrations, fasting plasma levels of C-peptide or insulin, while NEFAs increased during the initial 3 h post-acute subcutaneous GIP infusion (two to three times the normal physiological postprandial levels), possibly indicating increased AT lipolysis. Therefore, GIP appears to be playing a role in BAT thermogenic activity, which may have implications for the treatment of metabolic disorders. More work needs to be done to assess whether the observed increase in BAT activity post 6-day GIP infusion is sustained and whether GIP can induce lasting changes in thermogenesis of BAT.

### *In vitro/in vivo* inhibition of GIP

In 2002, the whole-body *Gipr*^−^^/−^ mouse model suggested that GIP is involved in obesogenic signaling pathways, as mice were found to be resistant to DIO when fed a HFD for >20 weeks (Miyawaki *et al.* 2002). Moreover, as previously discussed in the above sections, insulin receptor substrate-1 and GIPR appear to be reflective of insulin sensitivity. For example, under impaired insulin signaling, GIP increased fat uptake into adipocytes, and inhibiting insulin and GIP signaling reduced fat accumulation into adipocytes and promoted fat oxidation in the liver and skeletal muscle (Zhou *et al.* 2005). Nonetheless, these results remain unclear as the actions of GIP in the liver and skeletal muscle are known to be an indirect result of GIPR expression. Moreover, only subcutaneous and visceral WATs were examined in the *Gipr*^−/−^ mice until one study found a significant decrease in BAT weight and increase in BAT UCP1 mRNA levels in *Gipr*^−/−^ mice compared to WT mice fed a HFD (Hansotia *et al.* 2007). Interestingly, *Gipr*^−/−^ mice gain weight similar to WT mice when fed a HFD while being housed at thermal neutrality, indicating the effects of thermal stress in these mice (Furber *et al.* 2023). These findings suggest that inhibition of the GIPR in BAT may be important for regulating weight gain or systemic insulin sensitivity.

Surprisingly, male HFD-induced obese mice housed at room temperature lacking GIPR in their BAT (*Gipr*^BAT−/−^) showed no differences in fat and lean mass, body weight, food intake, basal energy expenditure, AT or organ weights compared to littermate controls (Beaudry *et al.* 2019). These findings suggest that BAT GIPR does not contribute to the lowering of body weight as observed in *Gipr*^−/−^ HFD-fed mice. However, fasted *Gipr*^BAT−/−^ mice exhibited elevated body temperature and maintained their temperature during an acute cold challenge, suggesting that these mice can still regulate their body temperature during cold exposure (Beaudry *et al.* 2019). Administering CL316, 243 (1 mg/kg), a β-3-adrenergic receptor agonist twice daily to *Gipr*^BAT−/−^ mice for 3 days showed no differences in energy expenditure, RER, or activity levels in mice, but increased lipid excursions during a lipid challenge, indicating impaired lipid tolerance when housed at room temperature. These mice lost body weight, increased BAT oxygen consumption, lowered BAT weight and adipocyte size, and improved lipid tolerance when housed in the cold for 12 weeks. From this work, it appears that inhibition of BAT GIPR in the cold is linked to fuel utilization, oxygen consumption, and thermogenic gene expression, rather than the regulation of body weight, fat, and/or lean mass. Furthermore, it is unknown if increases in endogenous GIP production impairs thermogenic function; however, this would appear to be unlikely as GIPR agonism promotes BAT thermogenic signatures (Samms *et al.* 2021).

When DIO mice were pre-treated with vehicle or mu-GIPR-Ab (25 mg/kg) for 24 h, and then with saline or GIP (D-Ala^2^-GIP; shorter-acting GIPR agonist compared to acyl-GIP), no changes in FA uptake in BAT were observed (Killion *et al.* 2020). Furthermore, [^3^H]triolein storage and lipolysis did not differ between *Gipr*^−/−^ and WT littermates in BAT when fed a HFD (Boer *et al.* 2021), suggesting that the absence of GIPR had no effect on fat storage or breakdown in BAT. Lastly, follow-up experiments showed no changes in stimulated or basal lipolysis in BAT in *ex vivo* experiments from HFD WT mice given GIP (200 and 1000 pM).

Together, these data suggest that the inhibition of GIPR in BAT is an interplay between various AT depots regulating energy utilization. They may explain changes in overall body weight when mice are provided excessive calories, i.e. HFD feeding or access to cold stimulation. GIPR activation and inhibition of GIPR signaling appears to act through different mechanisms in the BAT than in WAT, depending on housing and metabolic conditions; therefore, interpretation of the data needs to be carefully considered when assessing the effects on whole-body energy metabolism.

## Conclusion

The relationship between GIP and AT in metabolism, health, and disease is an important one that remains complex and is a rapidly evolving field of study, with future work further exploring the interplay of agonists and antagonists and examining potential sex-specific differences. The exact mechanisms of GIP on AT are not totally understood, as distinct depots present different actions of GIP and the adipogenic actions of GIP appear to differ based on the presence or absence of insulin, metabolic status of the individual (lean vs obese), and housing environments. More work is needed to determine the role of GIP at the level of the WAT and BAT to differentiate the metabolic actions of GIP from GLP-1 and glucagon. However, as co-agonist and tri-agonist GIPR-based therapies have shown reductions in body weight and improvement in lipid profiles in cellular, mouse, and human models, ongoing research opens many doors for understanding the underlying metabolic processes and developing therapies to address the health concerns of obesity and T2DM.

## Declaration of interest

The authors declare that there is no conflict of interest that could be perceived as prejudicing the impartiality of this review.

## Funding

This work was supported in part by various funds, including CIHR Project Grant 486421, Banting & Best Diabetes Centre; Novo Nordisk-BBDC Pilot and Feasibility Grant 2022-23, Novo Nordisk-BBDC New Investigator Award 2023-25, and Drucker Family Innovation Fundhttp://dx.doi.org/10.13039/100017413 Grant 2023-24. SK has received a fellowship from the Banting & Best Diabetes Centre-Novo Nordiskhttp://dx.doi.org/10.13039/501100004191 Graduate Studentship 2023-24. SL has received a fellowship from the Banting & Best Diabetes Centre, DH Gales Family Charitable Foundation Postdoctoral Fellowship 2022-23.
